# Automatic detection of single-electron regime and virtual gate definition in quantum dots using U-Net and clustering

**DOI:** 10.1038/s41598-026-38889-7

**Published:** 2026-02-14

**Authors:** Yui Muto, Michael R. Zielewski, Motoya Shinozaki, Kosuke Noro, Tomohiro Otsuka

**Affiliations:** 1 Research Institute of Electrical Communication, Tohoku University, 2-1-1 Katahira, Aoba-ku, Sendai, 980-8577 Japan; 2 Department of Electronic Engineering, Graduate School of Engineering, Tohoku University, Aoba 6-6-05, Aramaki, Aoba-Ku, Sendai, 980-8579 Japan; 3 Graduate School of Information Sciences, Tohoku University, 6-3-09 Aramaki-aza-Aoba, Aoba-ku, Sendai, 980-8579 Japan; 4 Unprecedented-scale Data Analytics Center, Tohoku University, 468-1 Aramaki-aza-Aoba, Aoba-ku, Sendai, 980-8572 Japan; 5 WPI Advanced Institute for Materials Research, Tohoku University, 2-1-1 Katahira, Aoba-ku, Sendai, 980-8577 Japan; 6 Center for Science and Innovation in Spintronics, Tohoku University, 2-1-1 Katahira, Aoba-ku, Sendai, 980-8577 Japan; 7 Center for Emergent Matter Science, RIKEN, 2-1 Hirosawa, Wako, Saitama 351-0198 Japan

**Keywords:** Semiconductor quantum dots, Machine learning, Auto-tuning, Quantum device scalability, Electronic devices, Nanoscale devices, Quantum information

## Abstract

To realize practical quantum computers, a large number of quantum bits (qubits) will be required. Semiconductor spin qubits offer advantages such as high scalability and compatibility with existing semiconductor technologies. However, as the number of qubits increases, manual qubit tuning becomes infeasible, motivating automated tuning approaches. In this study, we use U-Net, a neural network method for object detection, to identify charge transition lines in experimental charge stability diagrams. The extracted charge transition lines are analyzed using the Hough transform to determine their positions and angles. Based on this analysis, we obtain the transformation matrix to virtual gates. Furthermore, we identify the single-electron regime by clustering the Hough transform outputs. We also show the single-electron regime within the virtual gate space. These sequential processes are performed automatically. This approach will advance automated control technologies for large-scale quantum devices.

## Introduction

To advance quantum computing ^1^, the development of quantum bits (qubits) using various systems is progressing. Among these, semiconductor spin qubits are one of the promising candidates owing to their high scalability and compatibility with existing semiconductor technologies^2–8^. It is estimated that over one million qubits are required for practical quantum computers when using error correction like surface codes^9,10^. As the number of qubits increases ^11–13^, the parameter tuning process becomes complicated and infeasible, making automation critical for large-scale quantum computers^14–16^.

To form spin qubits, quantum dots that confine electrons within small regions are used. It is necessary to control the gate voltages that define the confinement potential of the dots to ensure that each dot contains one electron ^2,17^. One of the challenges in this process is an unintended interaction between quantum dots and gate electrodes. For example, while each gate voltage is designed to control the electron number within the quantum dot corresponding to its electrode, the electron number in neighboring quantum dots may also be affected. Such an unintended interaction complicates tuning, as the interdependency between gate voltages must be considered. To compensate for this crosstalk, the voltages of other gate electrodes must be adjusted simultaneously to isolate the effect to the target quantum dot. This is the core concept of a virtual gate^8,18^. These gates can be constructed by measuring the effect of the gate voltages on neighboring dots. This can be initially achieved by slightly varying the voltage of each gate electrode and analyzing the shift of the charge transition point^8^. However, this process is infeasible to perform manually for every gate electrode, especially in future large-scale qubits.

To address this issue, automated approaches have been developed to define virtual gates using image processing techniques^19–24^. Defining virtual gates requires determining the electrostatic coupling coefficients between each gate electrode and each quantum dot, also known as the sensitivity matrix. This, in turn, relies on accurately extracting the slopes of charge transition lines (CT-lines) in the charge stability diagram (CSD). A key technique for this purpose is the Hough transform, which automatically identifies straight lines, specifically CT-lines, in CSDs^25^. While the Hough transform requires binarized images, extracting CT-lines from images processed using common binarization methods, such as the Canny edge detection and Otsu’s methods, remains challenging. This difficulty arises through noise introduced by the device and experimental conditions ^26,27^, which persists through binarization, and leads to unexpected results when applying the Hough transform. Therefore, to obtain high-precision virtual gates using the Hough transform, a method is needed that is robust to noise and can accurately extract only the CT-lines within the CSDs.

With recent advancements in the field of machine learning, automated object detection methods have been developed and applied across various domains. One such method is U-Net^28^, a convolutional neural network architecture originally developed for biomedical image segmentation^29^, characterized by a symmetric encoder–decoder structure with skip connections. Its relatively simple architecture, composed primarily of convolutional, pooling, and upsampling layers, enables the effective extraction of key features even from limited amounts of training data. By training U-Net on annotated data of the target objects within images, precise automated segmentation can be achieved^30^.

**Fig. 1:**
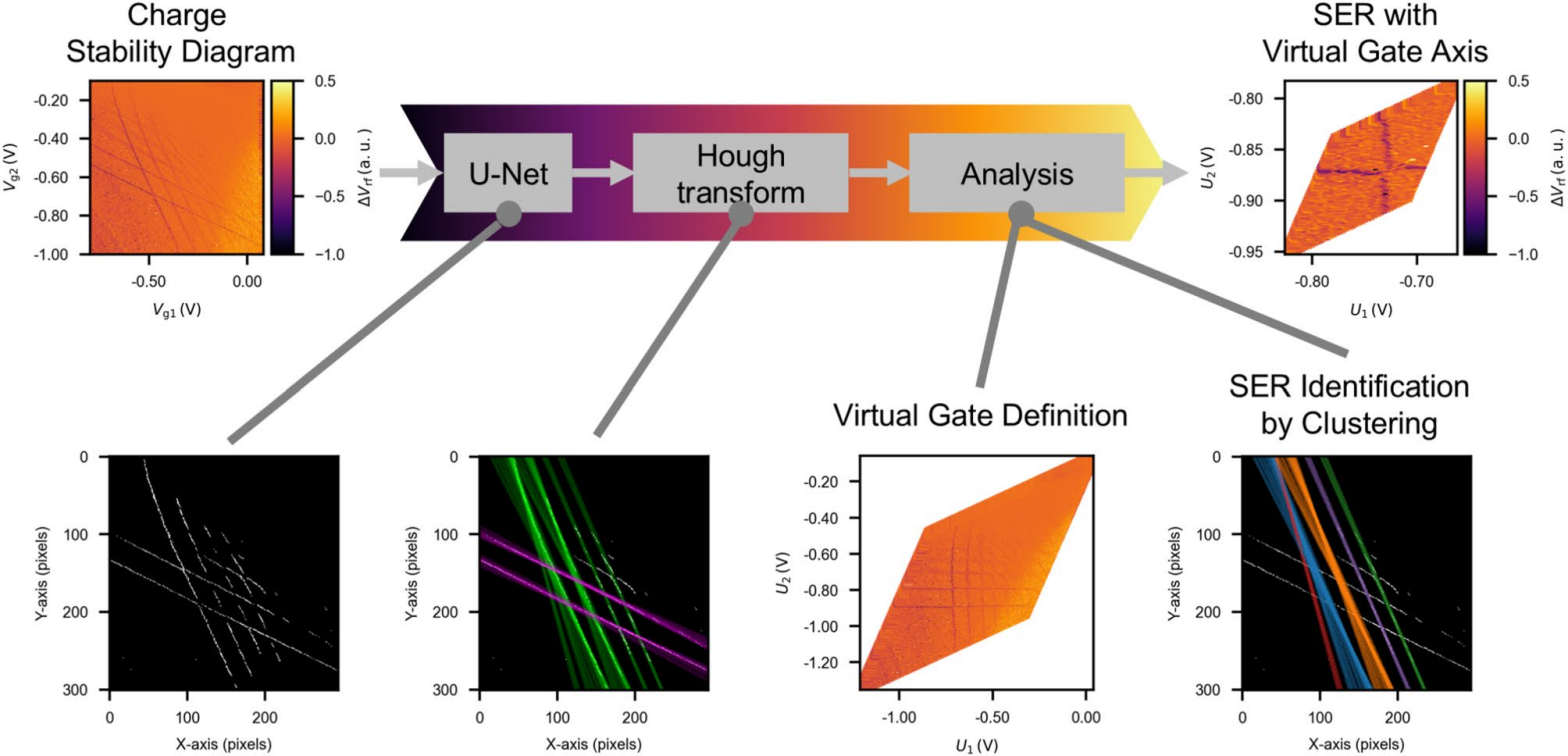
Framework of the proposed method. First, the CSD obtained from the measurement is used as input to the U-Net to extract transition lines. Next, the Hough transform is applied to detect straight lines. Based on the detected line information, virtual gate definition and SER identification through clustering are performed. Finally, the system automatically displays the SER with respect to the virtual gate axes.

In this study, we develop a method for the automatic definition of virtual gates and detection of the single-electron regime (SER). The framework of the proposed method is shown in Fig. 1. We employ a U-Net model to automatically detect CT-lines in CSD and apply the Hough transform to analyze these detected lines. Using this information, we define virtual gates. Additionally, we compare the accuracy of the binarization and Hough transform methods with alternative methods. We also identify the transition line positions using clustering and find the leftmost bottom crossing of the CT-lines adjacent to the SER, which is an important state for semiconductor spin qubits.

**Fig. 2:**
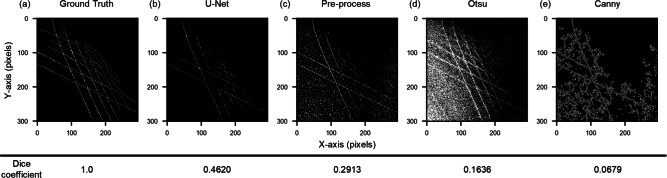
Binarized CSD images of (**a**) Ground truth and those processed by (**b**) U-Net, (**c**) Pre-process, (**d**) Otsu’s method, and (**e**) Canny edge detection. The values indicate the Dice coefficient of each method. The value closer to 1 indicates a higher similarity between the detection result and the ground truth.

## Results and discussion

### Accuracy verification of trained U-Net model

We apply the trained U-Net model to CSD of double quantum dots. The details of the training are described in the Methods section. The U-Net outputs pixel-wise segmentation probabilities for background and CT-lines, which are binarized by assigning the class with higher probability (1 for CT-lines, 0 for background). To compare with conventional methods, three other methods (pre-process, Otsu’s method, and Canny edge detection) are also tested. The pre-process method, proposed in our previous paper^31^, combines techniques such as Gaussian filtering to suppress noise in the CSD before binarizing the image. Otsu’s method^32,33^ is a well-known technique that automatically determines the threshold value for binarizing the image. While the Canny method^34^ typically requires manual threshold setting, it is commonly used as a binarization technique for edge detection before applying the Hough transform. We utilize the OpenCV package to apply both Otsu’s method^35^ and the Canny method^36^.

Figure 2a shows the manually annotated ground truth data. Specifically, an expert visually inspects the original measurement data, which is shown in Fig. 7b, and identifies CT-lines manually. It provides a reference for evaluating the segmentation performance of each method. Figure 2b–e display the results of binarization in the CSD using each method. The values shown below each figure indicate the Dice coefficient, which we use as a quantitative metric to compare our results with expert annotations (i.e., ground truth). A score closer to 1 indicates better alignment with the ground truth segmentation. As seen from these results, U-Net achieves the better alignment and extracts only CT-lines as binarized pixels, achieving a score of 0.4620. Regarding the validity of this value, we confirm that the entire method, including CT-line identification, virtual gate construction, and SER detection, which will be described later, operates reliably when the Dice score exceeds approximately 0.36, as detailed in Fig. S1 of the Supplementary material. Therefore, we consider this score sufficient for our method. In contrast, the other three methods (Fig. 2c–e) pick up experimental noise, reflecting low scores. This performance reflects the ability of U-Net to perform segmentation-based probabilistic classification, in which its skip connections allow simultaneous evaluation of both pixel-level features and global line continuity. By training on experimental data, we achieve robust classification even under noisy experimental conditions. Additionally, we present further simulations on the noise robustness of the U-Net model. Details of the simulation parameters and results can be found in Table S1 and Fig. S3 of the Supplementary material, while the noise dependence of the Dice score is presented in Fig. S2. Note that some CT-lines in the upper-right region of the CSD are not detected by U-Net. In this region, the signal is weak, making it difficult to distinguish CT-lines even for human experts. This could potentially be addressed by device and measurement developments such as techniques to maintain optimal charge sensor sensitivity ^37,38^.

**Fig. 3:**
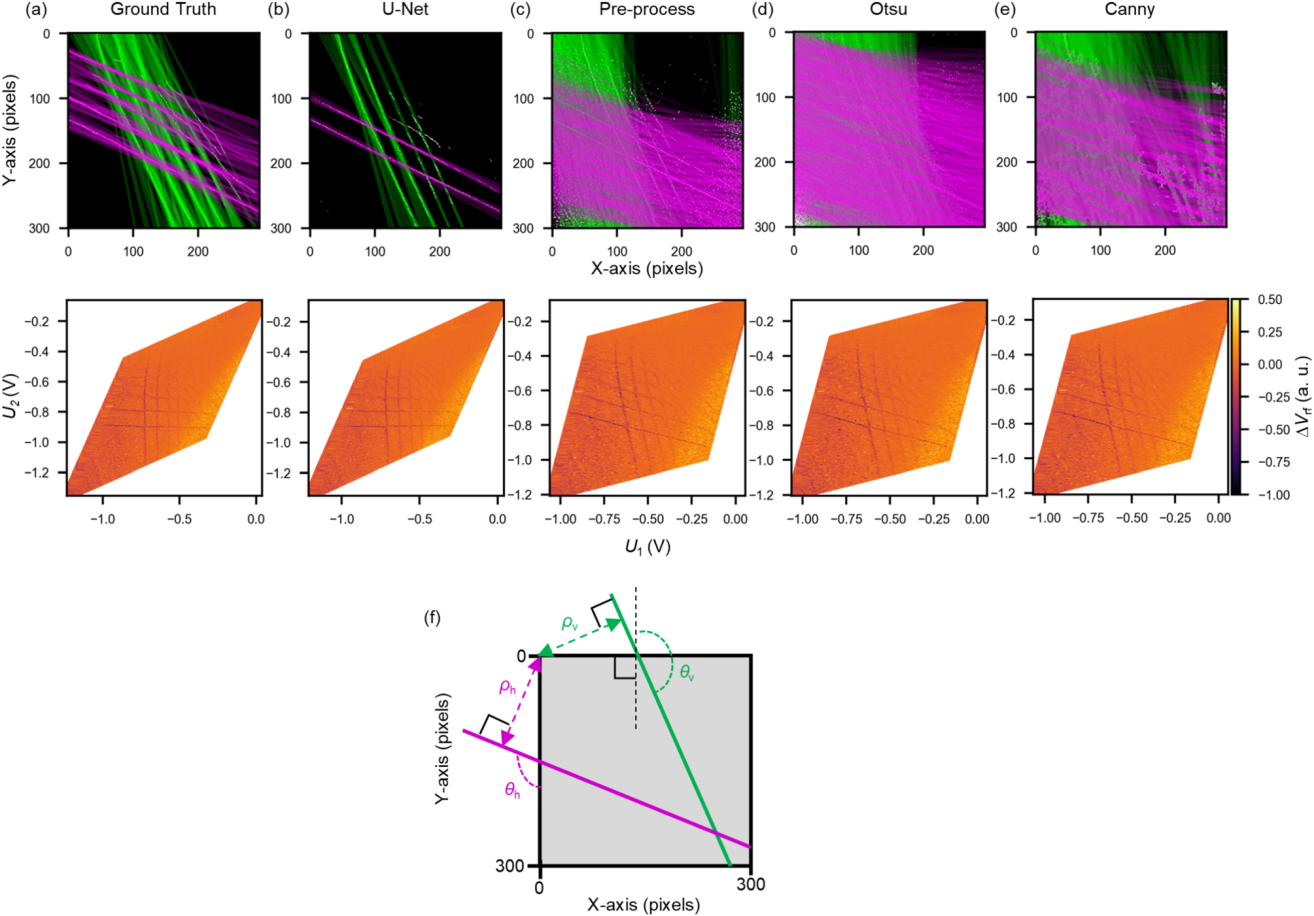
Detection of angle and position of CT-lines using the Hough transform for (**a**) ground truth, (**b**) U-Net, (**c**) pre-process, (**d**) Otsu’s method, and (**e**) Canny edge detection. Green and pink lines indicate vertical-like and horizontal-like CT-lines, respectively. Lower row shows charge stability diagrams with automatically defined virtual gate axes. (**f**) Schematic of the output parameters from the Hough transform.

### Hough transform and definition of virtual gate

The Hough transform is applied to the binarized CSD images obtained from each method to detect the angle and position of the CT-lines. These output parameters are used to automatically define the transformation matrix for virtual gates.

In a double quantum dot system, two types of CT-lines appear in the CSD: those that are nearly vertical and horizontal. In the case of multiple dot systems, additional CT-lines at different angles will appear. Virtual gates can reduce these CT-lines to two types by enabling independent control of the charge state in each dot.

The Hough transform is implemented using the OpenCV package^39^. The key parameters used in this study are summarized in Table 1.

**Table 1:** Parameters used in the cv2.HoughLines function.

Parameter	Vertical	Horizontal
rho	1	1
theta	$\pi /180$	$\pi /180$
threshold	30	30
min_theta	$5\pi /6$	$\pi /2$
max_theta	$\pi$	$2\pi /3$

Here, rho is the distance resolution of the accumulator in pixels, theta is the angle resolution of the accumulator in radians, threshold is the minimum number of votes required for a line to be considered as a valid straight line, min_theta is the minimum angle to check for lines, and max_theta is the upper bound for the angle. Specifically, we set the threshold to 30 points, taking into account the typical number of points that constitute a CT-line for the image size for which this code was designed. The Hough transform outputs the parameters $\rho$ and $\theta$ of the detected lines, where $\rho$ represents the perpendicular distance from origin to the line, and $\theta$ represents the angle formed by this perpendicular line and the horizontal axis measured in counter-clockwise.

We apply the Hough transform to the binarized CSD images from each method and show the results in the upper row of Fig. 3a–e. These figures are prepared by overlaying the straight lines detected by the Hough transform onto the binarized images shown in Fig. 2. The green lines represent the vertical-like CT-lines, and the pink lines represent the horizontal-like CT-lines. The same parameters for the Hough transform are used for all methods.

As seen in the upper row of Fig. 3a, accurately segmented CT-lines provide reliable angle and position values through the Hough transform.

Almost all of the CT-lines detected by U-Net are also captured by the Hough transform as shown in Fig. 3b. Note that some of the horizontal-like CT-lines are not detected. This is because unclear CT-lines constructed by a few pixels, such as those caused by sensor sensitivity issues, do not meet the threshold required by the Hough transform.

On the other hand, the Hough transform detects many lines other than CT-lines in the images binarized by other methods, as shown in the upper row of Fig. 3c–e. One of the possible scenarios is that sequential noise pixels are detected as CT-lines, as observed in Fig. 2c–e. Such misclassification due to noise pixels was also reported in a previous study^31^. While this issue can be mitigated by tuning the threshold parameter of the Hough transform for each method, our comparison across multiple threshold values showed that U-Net consistently provided the most robust performance (see Fig. S6 of the Supplementary material). This is likely because the binary images produced by U-Net contain less noise compared to those from other methods. Therefore, by leveraging U-Net for binarization, the cost of tuning the Hough transform threshold can be reduced, offering an advantage in terms of applicability to a wide range of experimental datasets.

To automatically define the virtual gate $\mathbf{U}$, we use the angle $\theta$ from the Hough transform. We define the virtual gate $\mathbf{U}$ using the mean angle $\theta$ calculated for both vertical-like and horizontal-like CT-lines, as follows:

1 $$\begin{aligned} \mathbf{U} = \mathbf{G} \cdot \mathbf{V}, \end{aligned}$$

2 $$\begin{aligned} \mathbf{U} = \begin{bmatrix} U_{1} \\ U_{2} \end{bmatrix}, \end{aligned}$$

3 $$\begin{aligned} \mathbf{G} = \begin{bmatrix} -\cos \theta _{\text {v}} & \sin \theta _{\text {v}} \\ -\cos \theta _{\text {h}} & \sin \theta _{\text {h}} \end{bmatrix}, \end{aligned}$$

4 $$\begin{aligned} \mathbf{V} = \begin{bmatrix} V_{\mathrm{g1}} \\ V_{\mathrm{g2}} \end{bmatrix}. \end{aligned}$$

Here, $\mathbf{V}$ is the physical plunger gate, $\theta _{\text {v}}$ is the mean angle $\theta$ of the lines detected as vertical-like CT-lines, and $\theta _{\text {h}}$ is the mean angle $\theta$ of the lines detected as horizontal-like CT-lines, as illustrated in Fig. 3f.

The CSDs transformed by virtual gates are shown in the lower row of Fig. 3. In the case of the ground truth data (Fig. 3a), it works well as the CT-lines appear at right angles. The virtual gates utilizing U-Net (Fig. 3b) display similar transformed CSDs to the ground truth because U-Net accurately extracts only the CT-lines from the CSD. For the other methods (Fig. 3c–e), the virtual gates do not work well. It is difficult to avoid the effect of additional noise lines on errors in $\theta _{\text {v}}$ and $\theta _{\text {h}}$, resulting in poorly defined virtual gates. These results show that U-Net can accurately detect CT-lines from noisy CSD data and is useful for automatically defining virtual gates through the Hough transform.

**Fig. 4:**
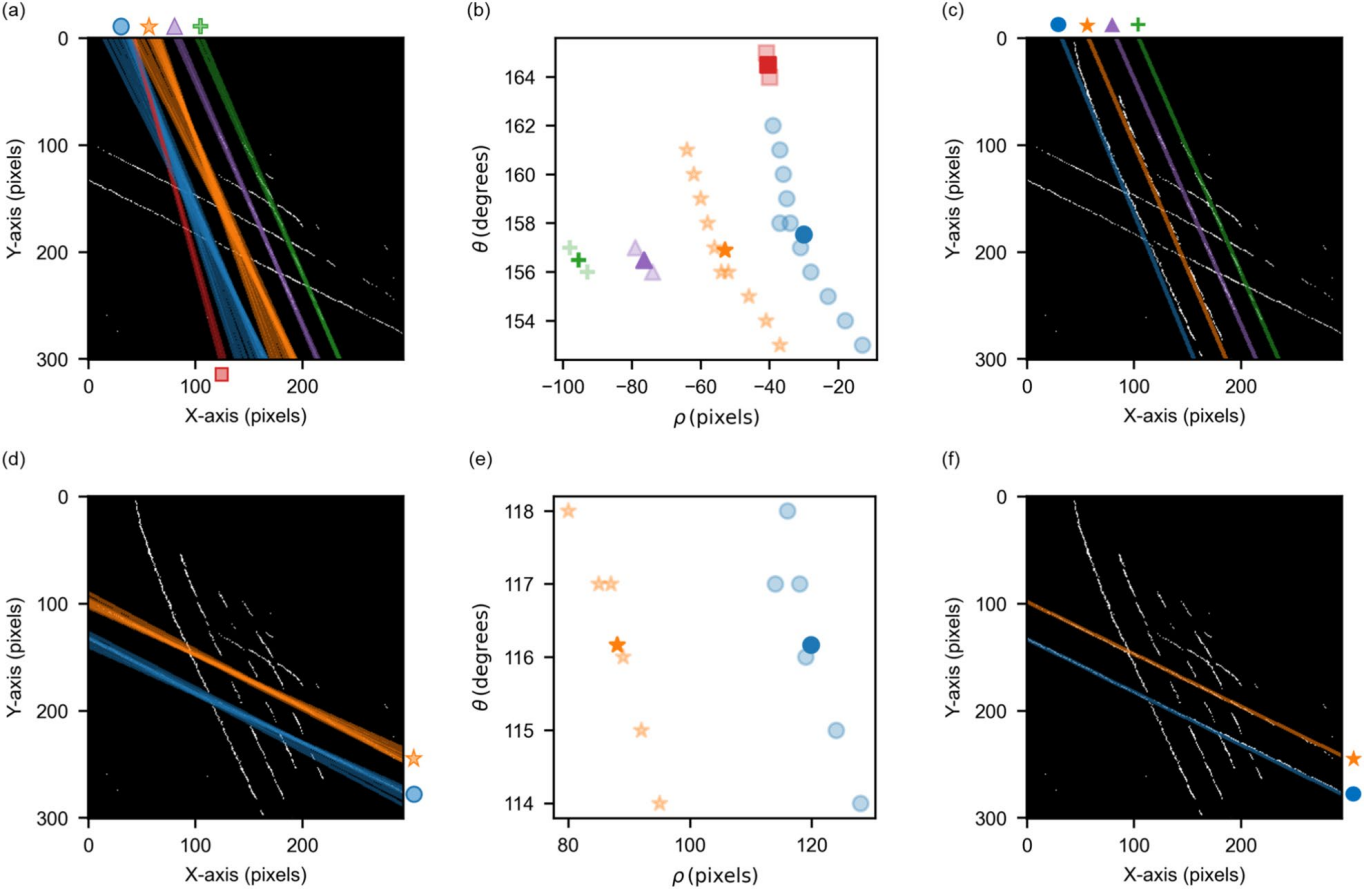
(**a**) Identification of vertical-like CT-lines by clustering. (**b**) Clustering results in the $\theta$-$\rho$ space, where each color corresponds to a cluster of detected CT-lines. (**c**) Merged lines obtained from multiple lines within each cluster. (**d**–**f**) Same analysis for horizontal-like CT-lines.

### Identification of CT-lines by clustering

As shown in Fig. 3b, the Hough transform detects multiple lines for a single CT-line, making it difficult to find SER although virtual gates can be defined. To address this issue, we apply clustering to the detected lines to identify each individual CT-line.

For the clustering process, we apply Density-based Spatial Clustering of Applications with Noise (DBSCAN)^40,41^. DBSCAN identifies clusters by grouping data points that are close to each other, enabling cluster detection even in complex scatter patterns. This method does not require specifying the number of clusters in advance, which is advantageous for automation. There is a certain sensitivity of the DBSCAN algorithm to the choices of hyperparameters and that careful choice of hyperparameters is helpful for optimal performance. We set min_samples to 1 so that all lines are included in some cluster. To ensure that the setting of eps works properly, it is crucial to first adjust the scales of $\rho$ and $\theta$. To accommodate data at different scales during clustering, we standardize $\rho$ to have a mean of 0 and a standard deviation of 1. Additionally, to match the scale of standardized $\rho$, we apply a weight of 14 to $\theta$ (in radians) and tune the value of the DBSCAN parameter eps accordingly, fixing it at 0.4 to align with the scaled features. The clustering results of $\rho$ and $\theta$ obtained from the Hough transform of vertical-like CT-lines are shown in Fig. 4a,b. The clustering successfully classifies most of the detected lines.

We identify each individual CT-line by taking the average of $\rho$ and $\theta$ in each cluster. The thick markers in Fig. 4b show these averages. Sometimes, the number of obtained clusters is greater than the actual CT-lines, resulting in an extra cluster and an averaged line (shown as red square markers in Fig. 4a,b). To remove this extra line, we use the following approach. Since CT-lines are nearly parallel to each other, they rarely intersect. Thus, if such an intersection is detected, it indicates duplicate detection of the same CT-line. In this case, we eliminate the extra line (the red marker) whose $\theta$ value deviates most from the mean $\theta$ of all lines.

As seen in Fig. 4c, the proposed clustering and merging process extracts all the CT-lines segmented by U-Net. This process also works well for horizontal-like CT-lines, as demonstrated in Fig. 4d–f.

**Fig. 5:**
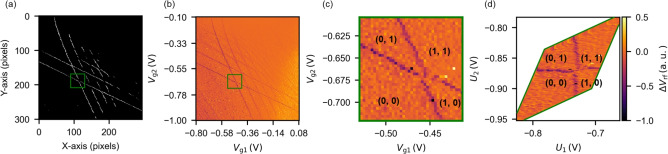
Automatically detected single-electron regime (green rectangle) in charge stability diagrams: (**a**) binarized map and (**b**) experimental data. (**c**) Zoomed-in view of single-electron region with gate voltage axes and (**d**) with virtual gate axes.

### Finding of the single electron regime in the charge stability diagram

Finally, we identify the SER in the CSD using the extracted CT-lines. We calculate the intersection point between the leftmost line (blue) in Fig. 4c and the bottommost line (blue) in Fig. 4f. This intersection corresponds to the leftmost bottom crossing of the CT-lines, marked by the automatically determined green rectangle in Fig. 5a. Figure 5b shows the experimental data with the marked SER region, and Fig. 5c provides a zoomed-in view of this region highlighted by the green rectangle. Here, (*n*, *m*) indicates the number of electrons in each quantum dot. Furthermore, we display this SER using virtual gates, as shown in Fig. 5d. These results demonstrate fully automated detection of CT-lines in CSD and finding SER with virtual gates.

**Fig. 6:**
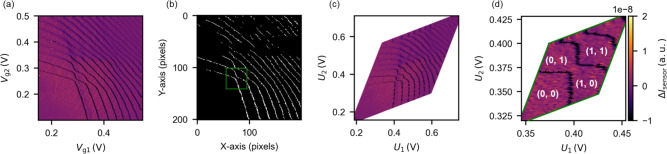
(**a**) Charge stability diagram from another group^42^ with differential processing along the horizontal axis. (**b**) Output of trained U-Net model with identified single-electron regime (green rectangle). (**c**) Charge stability diagram visualized with automatically defined virtual gates. (**d**) Single-electron regime represented in virtual gate space.

To validate the versatility of our approach, we apply our method to data from another group ^42^. Figure 6 shows the raw experimental data with differential processing along the horizontal axis and results of the processing flow we proposed. We also demonstrate the successful binarization of CSD using U-Net, definition of virtual gates, and finding of SER in CSD for another group’s data, showing the robust performance of our method.

To further evaluate the generalizability and robustness of our approach, we apply our method to two additional experimental CSDs from the same group ^42^. The results are presented in Figs. S4 and S5 of the Supplementary material. The results show that our method successfully identifies SER on high-resolution experimental CSDs measured over a wide gate voltage range. In the field of machine learning for semiconductor quantum dots, large-scale public datasets are still limited, and our current evaluation is based on a small number of examples. Nevertheless, we consider these results as preliminary evidence supporting the potential utility and broader applicability of our method. In future work, the performance on lower-resolution data may be improved by incorporating this type of data into the training set for the U-Net.

The advantage of our method lies in its time efficiency. It enables automatic definition of virtual gates and identification of SER, reducing tasks that previously took several minutes when performed manually to just approximately 0.5 s. This rapid automation is expected to be crucial for the future scaling and multiplexing of quantum dot systems.

## Conclusion

In this study, we demonstrate a method for the automatic definition of virtual gates and detection of the SER. We use U-Net to extract CT-lines from noisy CSD data and determine their angle and position by the Hough transform. This enables us to automatically define virtual gates. We also identify transition line positions using DBSCAN clustering and merging processes. Based on this approach, we demonstrate automatic display of SER in CSD with virtual gate axes. Furthermore, we apply our method to data from another group and confirm that it works well.

We also expect that the proposed approach is adaptable to various types of qubit systems. Specifically, since the CSD orientation for hole spin qubits is rotated by 180 degrees relative to that of electron spin qubits, we believe that this approach can be extended with a simple modification by identifying the SER through the intersection of the rightmost line and the topmost line after clustering.

We believe that our method will become increasingly valuable for operating large-scale quantum dot systems. In such systems, independent control of each quantum dot is essential. Our automated virtual gate approach enables this by allowing precise adjustment of the electron number in a selected double quantum dot, while maintaining the electron numbers in the surrounding dots. This capability enables the array to be partitioned into multiple double-dot pairs, allowing fully automated and independent SER searches for each. As a result, our method provides a scalable and systematic framework for achieving SER operation across the entire system, paving the way for large-scale quantum dot architectures.

**Fig. 7:**
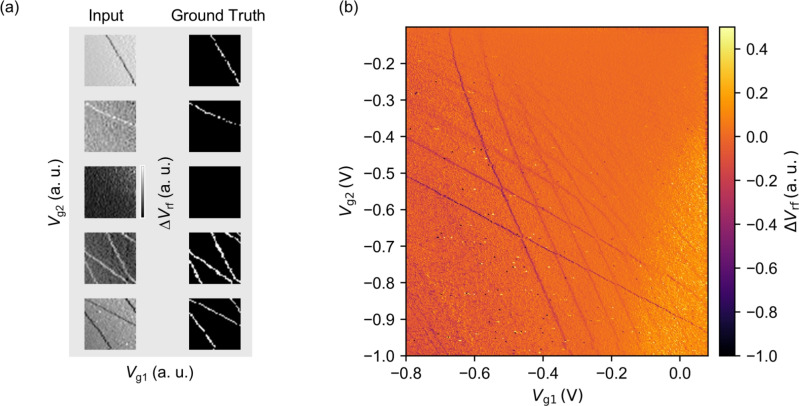
(**a**) Examples of training data pairs for the U-Net model, showing input data and corresponding ground truth (43 $\times$ 43 pixels). (**b**) A 301 $\times$ 295 pixel charge stability diagram used for evaluating the trained U-Net model, with differential processing along the horizontal axis.

## Methods

### Training of U-Net model

We describe the dataset used for training the U-Net model. Initially, we prepare 11 CSD images measured over a wide range of voltages from several different quantum devices.

Here, we employ data augmentation to construct a practical model even with a limited amount of original experimental data. The specific data augmentation methods used are “Crop”, “Random Invert”^43^, and “Random Adjust Gamma”. First, we apply “Crop” to extract multiple sub-images of size 48 $\times$ 48 pixels from each CSD image, thereby increasing the volume of the training samples. “Random Invert” randomly inverts image brightness to handle CT-lines that appear brighter or darker than the background, depending on the sensor’s operating point. “Random Adjust Gamma” randomly changes image contrast using gamma correction^44^ to improve the model’s robustness to variations in materials, device structures, and sensor sensitivity. The original experimental data already includes various noise levels, enabling the model to handle different noise conditions.

Through these processes, each raw image cropped from the CSD is independently augmented using “Random Invert” with invert probability of $p = 0.5$ and “Random Adjust Gamma”. In the Random Adjust Gamma operation, the gamma parameter $\gamma$ is varied uniformly from 0.25 to 4. These augmentations allow us to expand the original 11 images into a dataset with greater brightness and contrast variability, generating 182,101 training samples. These augmented images enable the model to adapt to diverse levels of contrast, brightness, and noise, and are subsequently downscaled to 43 $\times$ 43 pixels. Since sufficient performance is achieved using these augmented experimental images, no training with simulation data is performed. Figure 7a shows examples of the training data, where the left and right rows represent the input and ground truth data, respectively. 10% of the training samples are reserved for validation and not used for updating the model weights, in order to prevent overfitting during training.

In this study, we partially modify a pre-existing U-Net library^45^ and use it for our model training. We adopt the original U-Net architecture and implement the model in PyTorch (v.2.0.1). The hyperparameters are set as follows: a batch size of 64, a learning rate of 0.00001, and 100 epochs. Note that the actual number of epochs during training is 66 due to the early stopping to prevent overfitting.

The performance of the U-Net model is evaluated by the Dice coefficient. For a predicted result $\mathbf{X}$ and ground truth data $\mathbf{Y}$, the Dice coefficient $\textrm{Dice}(\mathbf{X}, \mathbf{Y})$ is defined as follows ^46^:

5 $$\begin{aligned} \textrm{Dice}(\mathbf{X}, \mathbf{Y}) = \frac{2 \sum _{i} X_i Y_i}{\sum _{i} X_i + \sum _{i} Y_i}. \end{aligned}$$

Here, $X_i$ and $Y_i$ represent the predicted and ground truth pixel values respectively, both taking binary values of 0 or 1. The Dice coefficient is a metric used to evaluate the overlap between the predicted result and the ground truth data, taking values between 0 and 1. A value closer to 1 indicates that the binarization result is similar to the ground truth.

The Dice loss, a loss function that should be minimized, is defined as follows:

6 $$\begin{aligned} \textrm{Dice Loss} = 1 - \textrm{Dice}(\mathbf{X}, \mathbf{Y}). \end{aligned}$$

The Dice loss is used to train the model to maximize the Dice coefficient, which increases the overlap between the predicted result and the ground truth. After each training epoch, we calculate the average Dice loss of the validation data over all batches. If there is no improvement for 5 consecutive epochs, training is stopped, and the model with the lowest Dice loss is selected.

The performance of the trained U-Net model is evaluated using the experimental data ^47^ shown in Fig. 7b. This figure is processed with horizontal differentiation and has a size of 301 $\times$ 295 pixels. Although the test data are significantly larger in size than the training data (43 $\times$ 43 pixels), U-Net handles the size discrepancy effectively, as shown in Fig. 2b. This is likely due to U-Net’s relatively simple architecture, which is composed primarily of convolutional layers and allows it to capture essential features even from a small amount of training data. This characteristic may contribute to its relatively good performance despite the large difference in image size between training and testing. Given that experimental settings may involve handling images of various sizes, robustness across different scales is highly beneficial.

## Supplementary Information

Supplementary Material 1

## Data Availability

The data that support the findings of this study are available upon reasonable request from the corresponding author.
