# Supplementary material for: Automatic detection of single-electron regime and virtual gate definition in quantum dots using U-Net and clustering
Source: Sci Rep. 2026 Feb 14;16:8161. doi: 10.1038/s41598-026-38889-7 (PMC12961048; doi:10.1038/s41598-026-38889-7)
Supplement: Supplementary file 1 — Supplementary Material 1 [file 41598_2026_38889_MOESM1_ESM.pdf]

Supplementary Information for:
Automatic detection of single-electron regime and virtual gate definition in quantum dots using U-Net and clustering

Yui Muto[1, 2], Michael R. Zielewski[3, 4], Motoya Shinozaki[5]
Kosuke Noro[1, 2], Tomohiro Otsuka[5, 1, 2, 6, 7]

[1]Research Institute of Electrical Communication, Tohoku University, 2-1-1 Katahira, Aoba-ku, Sendai 980-8577, Japan
[2]Department of Electronic Engineering, Graduate School of Engineering, Tohoku University, Aoba 6-6-05, Aramaki, Aoba-Ku, Sendai 980-8579, Japan
[3]Graduate School of Information Sciences, Tohoku University, 6-3-09 Aramaki-aza-Aoba, Aoba-ku, Sendai, 980-8579 Japan
[4]Unprecedented-scale Data Analytics Center, Tohoku University, 468-1 Aramaki-aza-Aoba, Aoba-ku, Sendai, 980-8572 Japan
[5]WPI Advanced Institute for Materials Research, Tohoku University, 2-1-1 Katahira, Aoba-ku, Sendai 980-8577, Japan
[6]Center for Science and Innovation in Spintronics, Tohoku University, 2-1-1 Katahira, Aoba-ku, Sendai 980-8577, Japan
[7]Center for Emergent Matter Science, RIKEN, 2-1 Hirosawa, Wako, Saitama 351-0198, Japan

1 Validity of the Dice score

To evaluate the required segmentation accuracy for the entire method, including CT-line identification, virtual gate construction, and Single-Electron Regime (SER) detection, to function properly, we investigate the minimum Dice score that ensures reliable operation. Specifically, we manipulate the Dice score by randomly inverting pixels in the transition-line regions of the Ground Truth (expert annotations) from the experimental dataset used in the main text. From this analysis, we observe that the method begins to function reliably when the Dice score exceeds approximately 0.36. An example of the output at this score is presented in Fig. S1.

Therefore, we conclude that segmentation accuracy corresponding to a Dice score of at least 0.36 is required for the overall method to work effectively. This also suggests that perfect segmentation is not necessary, as the subsequent processing steps, Hough transform, DBSCAN clustering, and line merging, are able to compensate for moderate segmentation errors.

2 Noise Robustness of U-Net at Different Noise Levels

We analyze the dependence of the dice score on noise levels using simulations with QArray [1, 2]. QArray allows for the addition of both white noise and telegraph noise to CSDs. Based on this capability, we prepare datasets by applying varying levels of white noise and telegraph noise to the

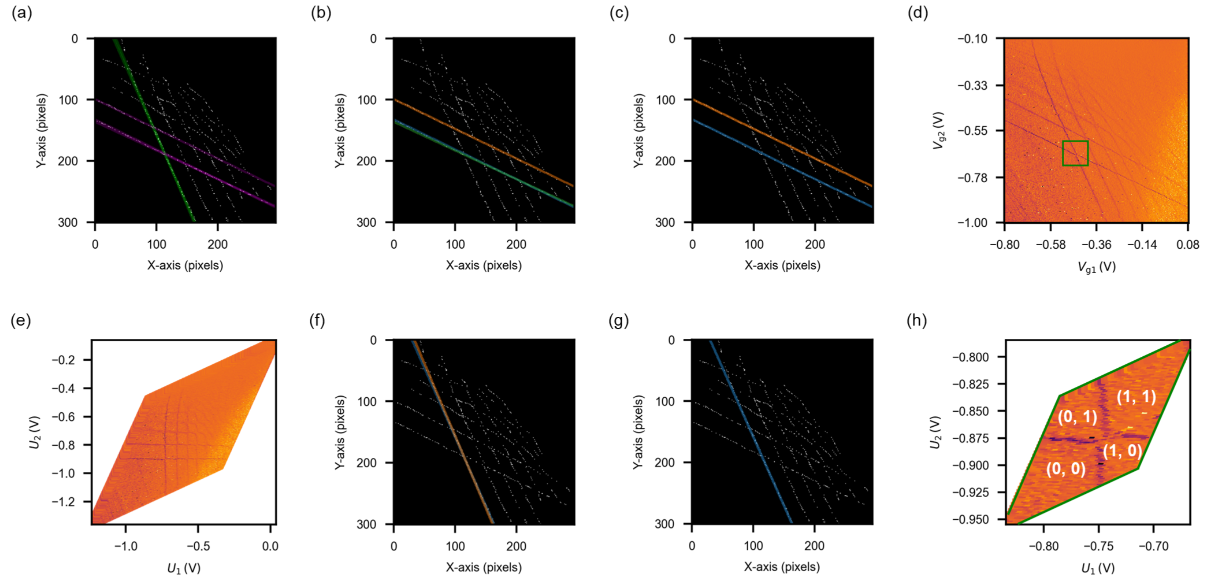

Figure S1: (a) Hough transform result for the data in which pixels corresponding to CT-lines in the original ground truth were randomly inverted to achieve a Dice score of 0.36. (b) Identification of horizontal-like CT-lines by clustering. (c) Merged lines obtained from multiple lines within each cluster. (d) Detected SER (green rectangle) in charge stability diagram (CSD). (e) CSD with automatically defined virtual gate axes. (f)-(g) Same analysis as (b)-(c) for vertical-like CT-lines. (h) Zoomed-in view of SER with virtual gate axes.

same CSD, using the parameters listed in Tab. S1, and evaluate how the Dice score of the U-Net changes under each condition. The results are shown in the colormap as shown in Fig. S2. In this colormap, the horizontal axis represents the `amplitude` parameter of the `WhiteNoise` class in the QArray simulation, which controls the intensity of the added white noise. The vertical axis corresponds to the switching probability parameters (`p01` and `p10`) of the `TelegraphNoise` class, which determine the transition probabilities between the two states of the telegraph noise.

As clearly seen in this figure, the white noise amplitude contributes more dominantly to the Dice score than telegraph noise. This is likely because white noise tends to obscure the transition lines as their amplitude increases. The Dice score remains stable when the white noise amplitude is below 6.3e-4, whereas it drops rapidly once the amplitude exceeds 1.6e-3.

An example of simulated data with a noise amplitude of 6.3e-4 and a telegraph noise probability of 1.6e-3 is indicated on the left side of Fig. S3, and the corresponding result of prediction by U-Net is shown on the right with green points.

Here, we discuss whether a typical measurement system can satisfy this threshold. We define the threshold of the signal-to-noise ratio (SNR) based on the sensor signal of 4.1×10^{-3} in our simulation, as described below.

$$\mathrm{SNR}_{\mathrm{thr}} = \frac{4.1 \times 10^{-3}}{6.3 \times 10^{-4}} \approx 6.51, \tag{1}$$

One of the typical SNR in an actual experiment is estimated using the data reported in the reference [3]. In this experiment, the sensor signal was approximately 0.42 mV. The noise corresponding to a standard deviation of σ_0 is 10 mV in a high-speed measurement using radio-frequency reflectometry. Considering the $\mathrm{SNR}_{\mathrm{thr}} = 6.51$, we estimate the required number of samples for signal integration based on experimental parameters. The high-speed measurement acquires data points

Table S1: Simulation parameters used in QArray

Class name	Parameters
`ChargeSensedDotArray`	`Cdd = [[0., 0.1], [0.1, 0.]],`<br>`Cgd = [[1., 0.6, 0.05], [0.4, 1., 0.05]],`<br>`Cds = [[0.02, 0.01]],`<br>`Cgs = [[0.04, 0.03, 1]],`<br>`coulomb_peak_width = 0.8, T = 100,`<br>`charge_carrier = 'electron'`
`ChargeSensedDotArray.`<br>`gate_voltage_composer.do2d`	`x_gate = 'P1',`<br>`x_min = -2, x_max = 6, x_res = 321`<br>`y_gate = 'P2',`<br>`y_min = -2, y_max = 6, y_res = 320`
`ChargeSensedDotArray.optimal_Vg`	`n_charges = [0.5, 0.5, 1.1]`
`TelegraphNoise`	`amplitude = 1e-2`

every 10 ns, and the effective SNR improves with the square root of the number of samples. To reach the $\mathrm{SNR_{thr}}$, approximately 2.4×10^4 samples are needed, corresponding to a total integration time of about 0.24 ms, which is fast enough to obtain CSDs. The white noise observed in radio-frequency reflectometry primarily originates from circuit components [4, 5], and can be considered independent of the quantum device. Considering these discussions, we believe that the proposed method can handle noise levels typically observed in actual experiments using various device architectures.

These results suggest that the proposed method has sufficient robustness under noise conditions typically observed in experimental data.

3 Additional Results on a Different Dataset

To further evaluate the generalizability and robustness of our approach, we apply it to two additional datasets not included in the main text. Both datasets were published by another research group [6]. The results obtained from these datasets are shown in Fig. S4 and Fig. S5, respectively. On these datasets as well, our method, consisting of U-Net segmentation, Hough transform, and clustering, works successfully and automatically displays the SER in the CSD along virtual gate axes.

4 Threshold Dependence of Hough Transform in each binarization method

In Fig. S6, we show the results of applying the Hough transform with various `threshold` values to the binarized images obtained from each method introduced in the main text (U-Net, Pre-process, Otsu, and Canny). Cells in which angle detection succeeds and the virtual gate axes are reasonably well defined are highlighted in pink. These results show that, although Pre-process and Canny perform well at certain threshold values, U-Net accommodates the widest range of thresholds. This is likely because the binary images produced by U-Net contain less noise compared to those from other methods. Therefore, by leveraging U-Net for binarization, the cost of tuning the Hough transform `threshold` can be reduced, offering an advantage in terms of applicability to a wide range of experimental datasets.

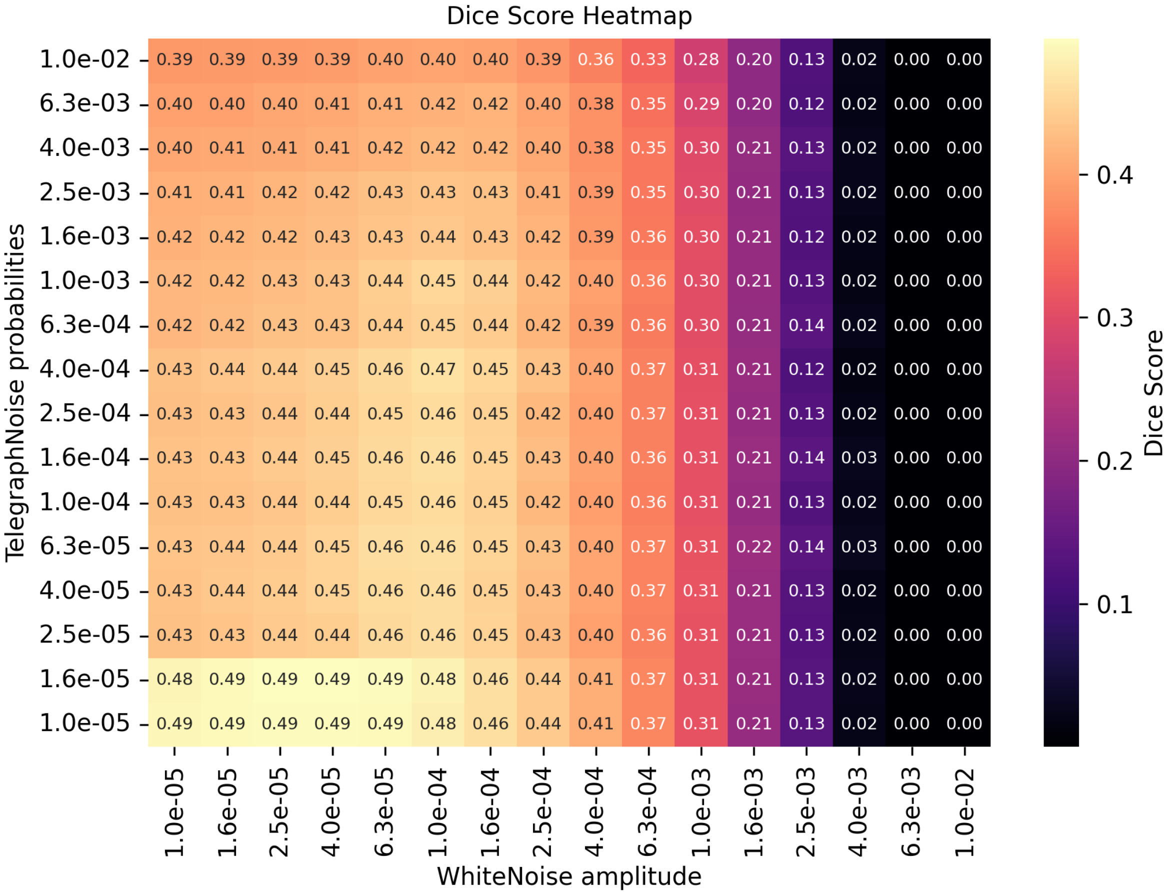

Figure S2: Colormap showing the dependence of the model output on simulation data with added white and telegraph noise, as generated by the QArray framework. The horizontal axis represents the `amplitude` parameter of the `WhiteNoise` class, and the vertical axis corresponds to the switching probability parameters `p01` and `p10` of the `TelegraphNoise` class. The color bar indicates the Dice score, which serves as a measure of model performance. The numerical value of the Dice score is also displayed in each cell of the colormap.

5 Implementation Details

The U-Net model was trained using an NVIDIA GeForce RTX 3070 GPU. The total training time was approximately 4 hours.

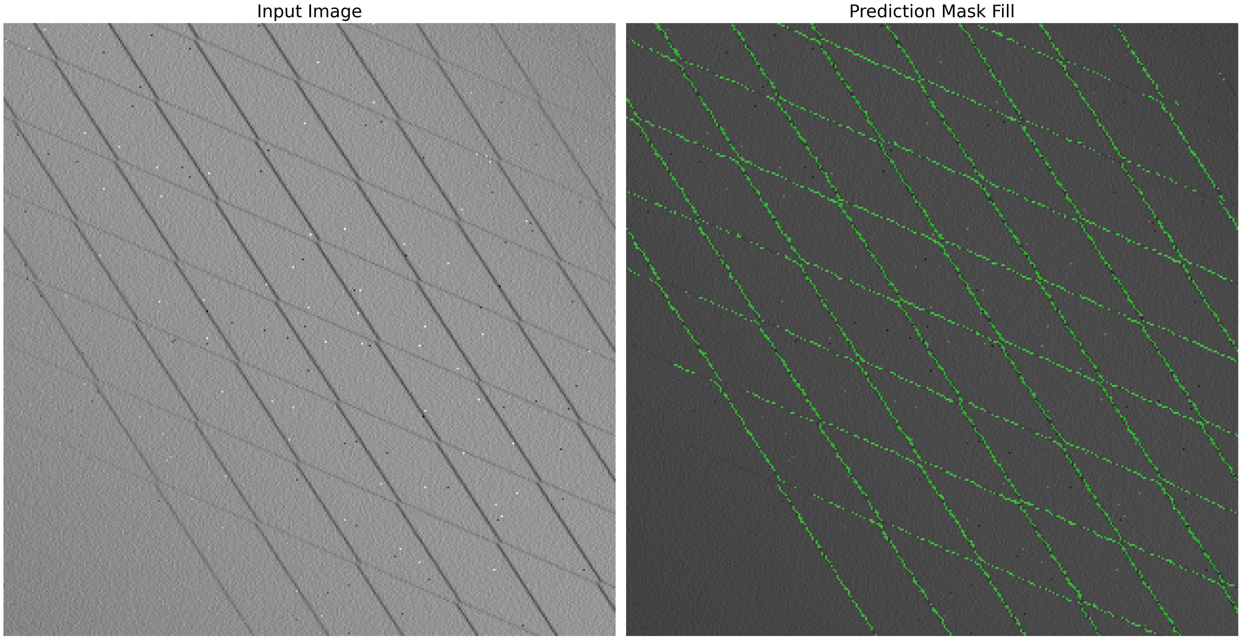

Figure S3: Simulation data generated by QArray with a white noise `amplitude` parameter of 6.3e-4 and telegraph noise switching probabilities `p01` and `p10` both set to 1.6e-3.

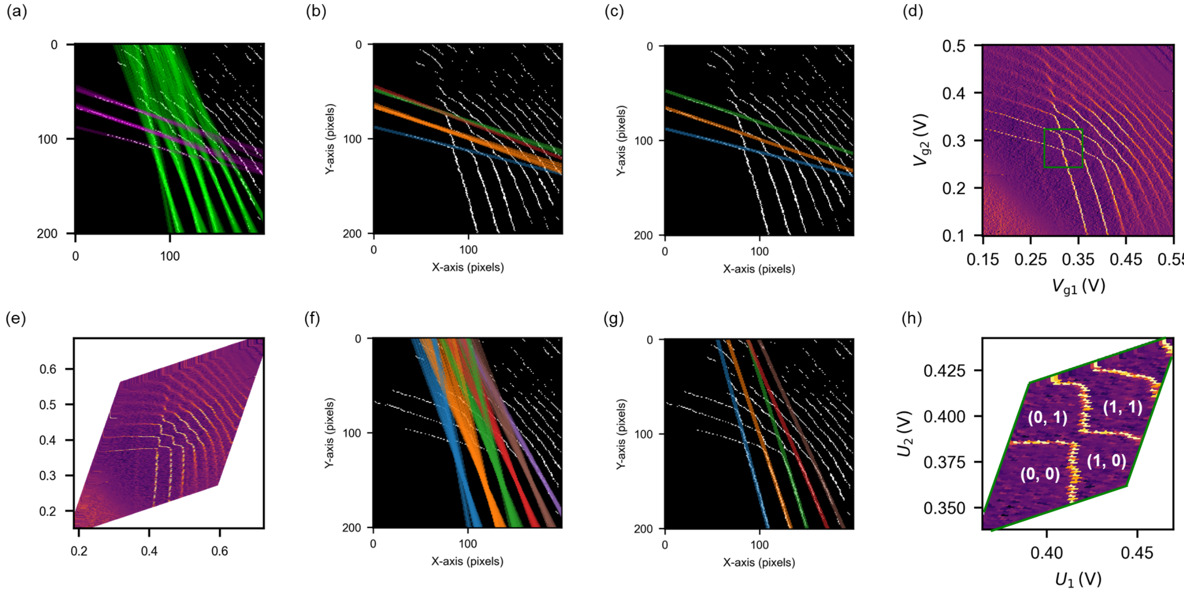

Figure S4: Results for the first additional dataset [6]. (a) Hough transform result. (b) Identification of horizontal-like CT-lines by clustering. (c) Merged lines obtained from multiple lines within each cluster. (d) Detected SER (green rectangle) in CSD. (e) CSD with automatically defined virtual gate axes. (f)-(g) Same analysis as (b)-(c) for vertical-like CT-lines. (h) Zoomed-in view of SER with virtual gate axes.

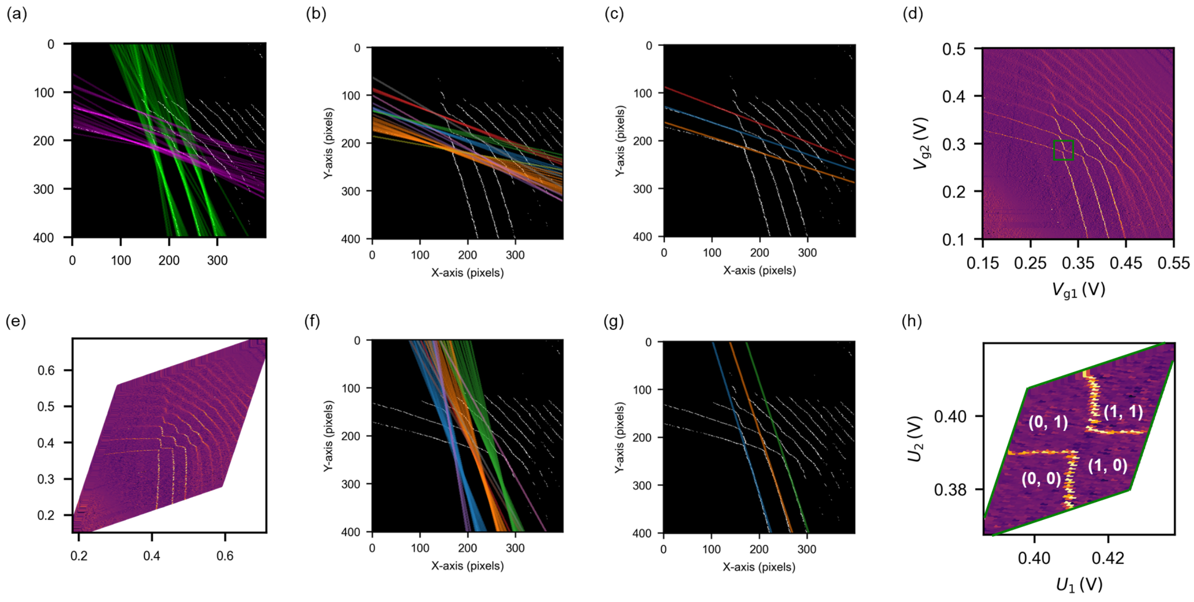

Figure S5: Results for the second additional dataset [6]. Same analysis steps as in Fig. S4.

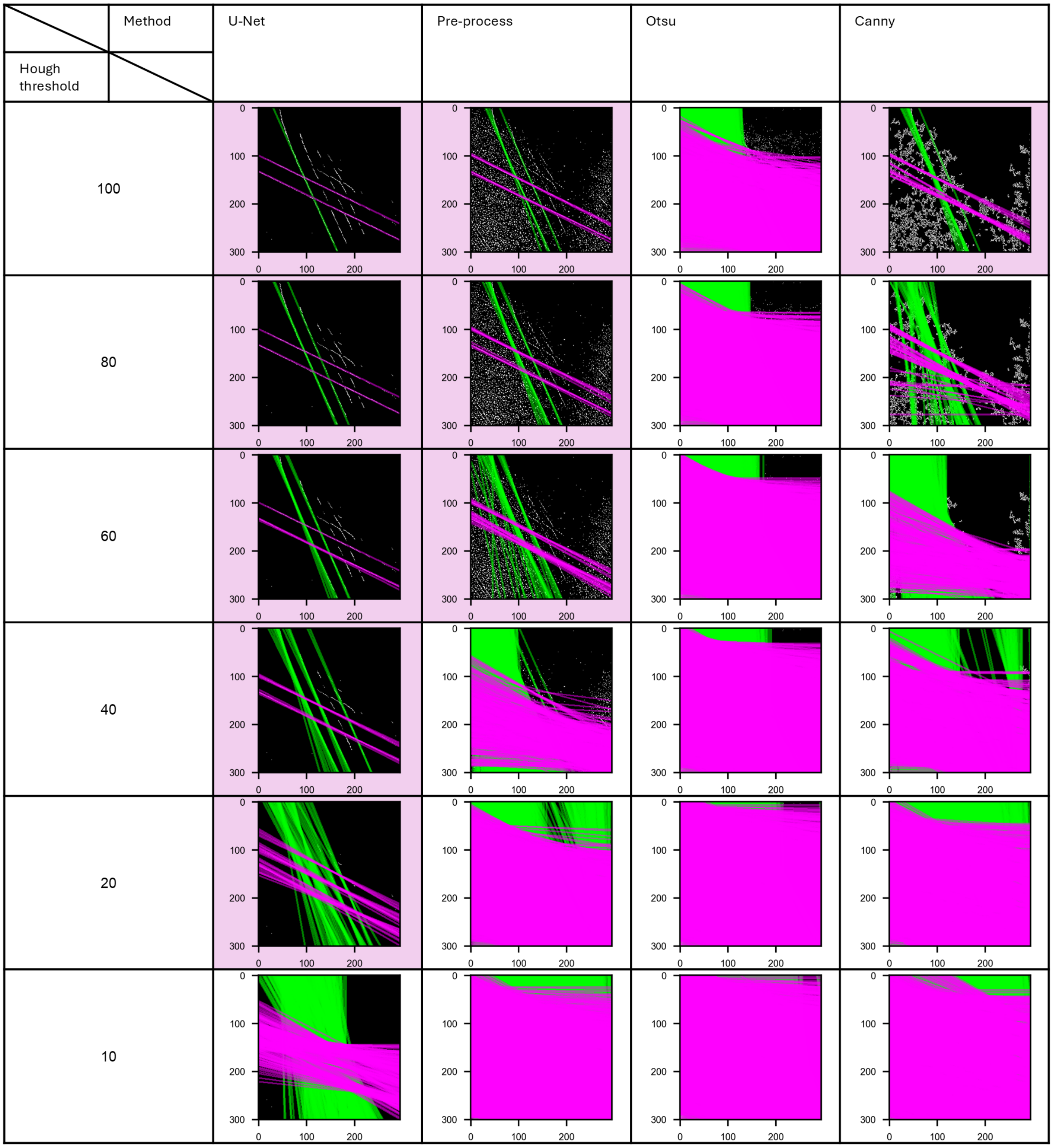

Figure S6: Results of applying the Hough transform with multiple values of the parameter `threshold` to the binarized images produced by each method. Cells in which angle detection succeeded and the virtual gate axes were reasonably well defined are highlighted in pink.

References

[1] van Straaten, B., Hickie, J. & Carlsson, C. Qarray. https://github.com/b-vanstraaten/qarray (2021). Accessed: 2025-07-28.

[2] van Straaten, B. *et al.* Qarray: a gpu-accelerated constant capacitance model simulator for large quantum dot arrays. *arXiv preprint arXiv:2404.04994* (2024).

[3] Noro, K. *et al.* Charge sensing of few-electron zno double quantum dots probed by radio-frequency reflectometry. *arXiv preprint arXiv:2501.04949* (2025).

[4] Shinozaki, M. *et al.* Gate voltage dependence of noise distribution in radio-frequency reflectometry in gallium arsenide quantum dots. *Applied Physics Express* **14**, 035002, DOI: 10.35848/1882-0786/abe41f (2021).

[5] Shinozaki, M., Muto, Y., Kitada, T. & Otsuka, T. Charge-state estimation in quantum dots using a bayesian approach. *Phys. Rev. Appl.* **23**, 034078, DOI: 10.1103/PhysRevApplied.23.034078 (2025).

[6] Zwolak, J. P., Ziegler, J., Kalantre, S. S. & Taylor, J. M. QFlow 2.0: Quantum dot data for machine learning (2022). National Institute of Standards and Technology, https://doi.org/10.18434/T4/1423788.
